# A role for Ras signaling in modulating mammalian aging by the GH/IGF1 axis

**DOI:** 10.18632/aging.100309

**Published:** 2011-04-19

**Authors:** João Pedro de Magalhães

**Affiliations:** Integrative Genomics of Ageing Group, Institute of Integrative Biology, University of Liverpool, Liverpool, UK

The discovery that mutations in single genes can modulate aging was not only fascinating but it provided researchers with animal models with which to study aging in cohorts of different aging rates [1-2]. According to the GenAge database [3], at the time of writing, genetic manipulations in 68 genes have been shown to affect lifespan in mice. Among mouse genes in which mutations extend lifespan, a number are part of the growth hormone/insulin-like growth factor 1 (GH/IGF1) axis. As such, it is widely acknowledged that decreased GH/IGF1signaling in mice can extend lifespan, while increased GH levels may accelerate aging [4-5]. Although the GH/IGF1axis is one of the major pathways regulating aging in mammals, its precise downstream mechanisms remain a subject of debate and scrutiny.

In this year's March issue of AGING, Borras et al. show that mice deficient in Rasgrf1, a guanine nucleotide–releasing factor for Ras, exhibit a significant increase (~20%) in average and maximum lifespan. This increase in lifespan was independent of cancer mortality and was accompanied by a retardation in age–related functional and physiological decline [6]. Ras homologues have been associated with lifespan modulation in yeast [7], so it seems that Ras signaling is another example of an evolutionary conserved pathway modulating aging. Rasgrf1 is unique among Ras family members, however, as it is only expressed in a few specific tissues, in particular in pancreatic β–cells and in some regions of the brain. Thus far, Rasgrf1 has been primarily associated with learning and memory [6,8-9].

Because of its expression in the brain, Rasgrf1 has been shown to regulate the synthesis and release of GH, and previous studies showed that Rasgrf1 deficient mice have lower levels of GH [9-10]. In line with these previous studies, and given that GH impacts on IGF1 levels, Rasgrf1 deficient mice showed lower IGF1 levels [6]. Decreased GH/IGF1 signaling results in stunted growth and reduced adult body size, which has been associated with life-extension in numerous models [11]. Borras et al. and other authors also observed that Rasgrf1deficient mice are smaller than controls [6,9-10]. Therefore, it is likely that decreased GH/IGF1 signaling is the mechanism by which Rasgrf1 impacts on aging. Changes in GH/IGH1 have also been hypothesized to be related to the mechanisms of caloric restriction [4-5], which is in line with the observation by Borras et al., that Rasgrf1 deficient mice have metabolic profiles similar to mice under caloric restriction.

Because Rasgrf1 mutants have altered GH/IGF1 signaling, I had predicted that they would be long-lived [12]. Moreover, it was reported that bi-maternal mice are long-lived and, because Rasgrf1is an imprinted gene expressed from the paternal allele in neonates, it is possible that bi-maternal mice are long-lived due to Rasgrf1 effects [12-13]. Although conducted in different labs and strains, bi–maternal mice had a similar increase in average lifespan (28%), though a more modest increase in maximum lifespan (~5%).

Rasgrf1 deficient mice are therefore another model to study the modulation of aging by the GH/IGF1 axis. Interestingly, mice expressing Rasgrf1from both alleles have increased body size and higher IGF1 levels [9]. Although the lifespan of these animals is unknown, they could be seen as a complementary model to the Rasgrf1 knock-outs. Consequently, since they are available with both increased GH (biallelic Rasgrf1 mice) and decreased GH levels (Rasgrf1 knock outs), the different Rasgrf1 mutant mice may be an excellent new model to study aging and its modulation by the GH/IGF1 axis.
